# Conserving evolutionary history does not result in greater diversity over geological time scales

**DOI:** 10.1098/rspb.2018.2896

**Published:** 2019-06-05

**Authors:** J. L. Cantalapiedra, T. Aze, M. W. Cadotte, G. V. Dalla Riva, D. Huang, F. Mazel, M. W. Pennell, M. Ríos, A. Ø. Mooers

**Affiliations:** 1 Museum für Naturkunde, Leibniz-Institut für Evolutions und Biodiversitätsforschung, Invalidenstraße 43, Berlin 10115, Germany; 2 Departamento de Ciencias de la Vida, Universidad de Alcalá, 28805 Alcalá de Henares, Madrid, Spain; 3 School of Earth and Environment, The University of Leeds, Leeds LS2 9JT, UK; 4 Department of Biological Sciences, University of Toronto-Scarborough, 1265 Military Trail, Toronto, Ontario, Canada M1C 1A4; 5 Department of Ecology and Evolutionary Biology, University of Toronto, 25 Wilcocks Street, Toronto, Ontario, Canada M5S 3B2; 6 Department of Statistics, University of British Columbia, 4200-6270 University Boulevard, Vancouver, BC, Canada V6T 1Z4; 7 Department of Botany, University of British Columbia, 4200-6270 University Boulevard, Vancouver, BC, Canada V6T 1Z4; 8 Department of Zoology, University of British Columbia, 4200-6270 University Boulevard, Vancouver, BC, Canada V6T 1Z4; 9 School of Mathematics and Statistics, University of Canterbury, Private Bag 4800, Christchurch 8140, New Zealand; 10 Department of Biological Sciences and Tropical Marine Science Institute, National University of Singapore, 16 Science Drive 4, Singapore 117558, Singapore; 11 Department of Biological Sciences, Simon Fraser University, 8888 University Drive, Burnaby, British Columbia, Canada V5A 1S6; 12 Departamento de Paleobiología, Museo Nacional de Ciencias Naturales (CSIC), José Gutiérrez Abascal 2, 28006 Madrid, Spain

**Keywords:** conservation, phylogenetic diversity, diversification, macroevolution

## Abstract

Alternative prioritization strategies have been proposed to safeguard biodiversity over macroevolutionary time scales. The first prioritizes the most distantly related species—maximizing phylogenetic diversity (PD)—in the hopes of capturing at least some lineages that will successfully diversify into the future. The second prioritizes lineages that are currently speciating, in the hopes that successful lineages will continue to generate species into the future. These contrasting schemes also map onto contrasting predictions about the role of slow diversifiers in the production of biodiversity over palaeontological time scales. We consider the performance of the two schemes across 10 dated species-level palaeo-phylogenetic trees ranging from Foraminifera to dinosaurs. We find that prioritizing PD for conservation generally led to fewer subsequent lineages, while prioritizing diversifiers led to modestly more subsequent diversity, compared with random sets of lineages. Importantly for conservation, the tree shape when decisions are made cannot predict which scheme will be most successful. These patterns are inconsistent with the notion that long-lived lineages are the source of new species. While there may be sound reasons for prioritizing PD for conservation, long-term species production might not be one of them.

## 1. Introduction

Given our limited resources for preserving biodiversity during the current extinction crisis [1,2], arguments have been made for protecting sets of more distantly related species from extinction as one principle for triage. The belief is that sets of distantly related species will have a wider variety of traits, and as a result will (i) produce higher-functioning ecosystems [3,4], (ii) have a greater ability to contribute to benefits to humans under changing and uncertain future environments in the medium term [5–7], and (iii) harbour greater evolutionary potential for future lineage-specific adaptation [8,9] and diversification over longer time scales [10], ensuring that biodiversity and the benefits from (i) and (ii) exist into the future. If one considers lineage accumulation as one measure of such diversification, this third argument for preserving sets of distantly related lineages can be tested.

All these arguments above have been used to support conservation strategies that prioritize total evolutionary history, which is achieved by focusing on sets of species that are distantly related [8] and typically select for slow diversifiers. In the context of argument (iii), there is, however, an alternative strategy, which is to conserve the ongoing diversification process *per se* [11–16]. This scheme would prioritize sets of rapidly speciating clades, such that sets of closely related taxa would be protected instead of distantly related ones.

These alternative strategies also map onto a longstanding question in palaeobiology, namely whether long-lasting lineages are statistically unremarkable (e.g. due to the age-independence of macroevolutionary processes) [17,18], are phenotypically average and repeatedly act as ‘biodiversity begetters’ [19,20], or are dead ends [21]. Because species on long terminal branches contribute more to phylogenetic diversity (PD) on average [22], the PD-maximizing strategy is predicated on the expectation that such lineages are more likely to speciate in the future than the average lineage; the speciation rate-maximizing strategy is predicated on the diversification potential of long-lived lineages being lower than average.

**Figure 1.**
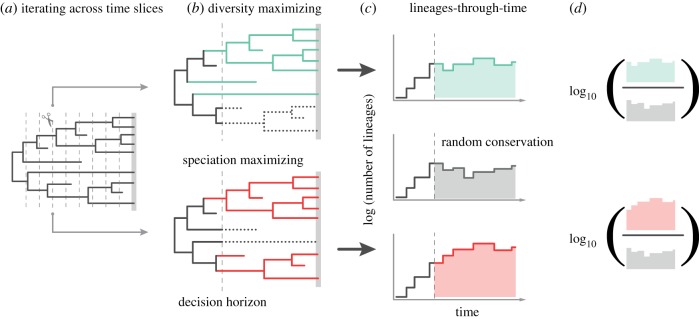
Schematic workflow. For each tree, we set a series of time slices (dashed lines in (*a*)) at which we conserve a proportion of lineages (here in colours) based on diversity (PD-maximizing) or speciation rate (DR-maximizing) strategies (coloured lineages) (*b*). Each conservation strategy yields a subsequent diversity trajectory (*c*) represented by a lineages-through-time curve. These curves can be compared with the diversity trajectory obtained if the same number of lineages were conserved randomly (grey curve in (*c*)). At each time slice, the performance of the PD (or DR) maximizing algorithms is calculated (*d*) as the (logarithm of) the area under the diversity curve following a conservation strategy divided by the area under the diversity curve produced from random conservation. (Online version in colour.)

**Figure 2.**
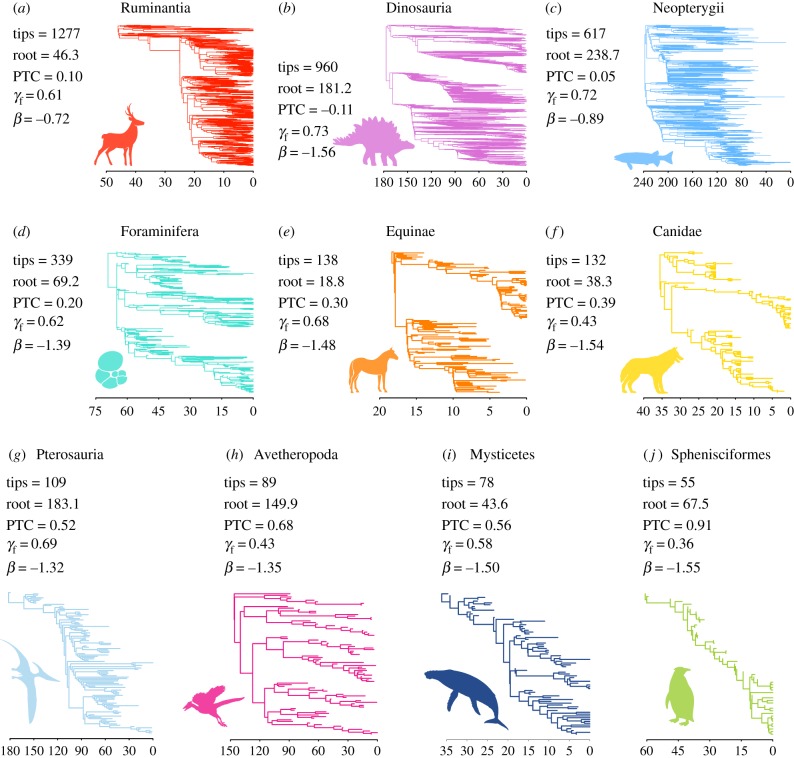
Datasets. The 10 fossil-rich phylogenetic datasets used in this study, ordered from largest to smallest. We report a series of tree metrics: the number of tips, the maximum root age over 100 trees from the posterior distribution of trees (in millions of years), average phylo-temporal clustering (PTC, the extent to which temporally synchronous divergence and extinction events are also phylogenetically clustered; see Material and methods and electronic supplementary material, figure S1), fossil *γ* (*γ*_f_, the proportion of tree length held by the branches leading to the tips) and tree balance (*β*, the extent to which subclades in the tree are the same size). Note that the temporal axis below the trees reflects node depth and not necessarily absolute age. (Online version in colour.)

Predicting the future is difficult, and so it is unclear which approach is more likely to ensure more biodiversity in the future [15]. The time-horizons over which we expect such actions to have effects are so long that it is difficult to compare the two scenarios experimentally. As an alternative, we can look to the past [23] and ask a simple question: which conservation strategy implemented millions of years ago would have resulted in greater species richness over subsequent geological time scales? We perform this ‘palaeo-conservation’ experiment by (i) mimicking conservation choices at different time points in history using dated, species-level fossil phylogenetic trees that include many extinct taxa and then by (ii) replaying the tape of life to ask whether targeted conservation decisions based on phylogeny at those past time points would have led to more species over the subsequent course of evolution compared to randomly selecting taxa, where random choice captures the many prioritization decisions that do not consider past diversification. We query our palaeo-phylogenies at an arbitrary time (a time slice) and model the sorts of decisions we face now by retaining sets of distantly related lineages (using Faith's measure of PD as our metric) [8,10], sets of rapidly speciating lineages (using Jetz *et al*.'s ‘species-level diversification rate’, or DR, as our metric) [24,25] and random sets of lineages, discarding unchosen lineages as victims of external extinction drivers. We then ask whether the conserved set leads to more or less subsequent biodiversity than the random set, measured as the total number of lineages through time. We repeat this procedure over many sequential time slices (either every million years or every 5 million years; see Material and methods) to ask which of the two strategies (PD-maximizing or speciation-rate-maximizing), if either, is on average better than the random approach. Our workflow is presented in figure 1, and our dataset of 10 dated phylogenies is presented in figure 2 and electronic supplementary material, table S1.

This experimental test of the effects of palaeo-conservation requires three linked assumptions. Almost all our palaeo-phylogenies are incomplete, and we assume that their shapes capture general macroevolutionary processes rather than biased sampling [26]. Because we are pruning lineages in the past and then looking at subsequent species richness, we also assume that lineage interactions are not overriding drivers of realized diversification. Finally, we assume that the shapes of these palaeo-phylogenies are representative of the unknown complexities of future long-term macroevolution (e.g. that past processes such as changing biogeographic theatres, changing productivities and the appearance of future key innovations that have led to the tree shapes we sample represent these processes into the future). Overall, we hope that the shapes of these real palaeo-phylogenies capture actual process better than would simulated phylogenies.

Given these assumptions, the simple exercise we present should help us evaluate arguments for considering macroevolution as a driver of conservation prioritization. As we show below, maximizing PD offers no diversification returns across our 10 trees, while maximizing speciation rate could. And, while overall tree shape did predict when sets of distantly related lineages would have led to more biodiversity, tree shape measured at any focal time slice when decisions are made does not.

## 2. Results and discussion

**Figure 3.**
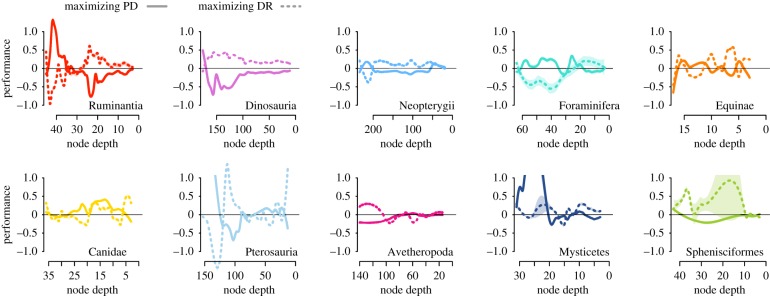
Performance at 30% conservation through time. The PD-based strategy is depicted with a solid line and the DR-based strategy with the dotted line. We show a LOESS fit to the data over 100 trees (except for Foraminifera), where the *x*-values are the times of the time slices in millions of years, and the *y*-values are (the log_10_ of) the area under the median LTT plot from our PD-maximizing strategy divided by the area obtained under the median LTT from random conservation. The thin horizontal line (at *y* = 0) represents the value below which random choice outperforms each conservation strategy. An optimal smoothing parameter for the LOESS fit was selected using an Akaike information criterion [27] so that the resulting curve captures the general trend, and reduces influence of extreme points. Shaded areas represent the 95% confidence interval of the LOESS fit. (Online version in colour.)

**Figure 4.**
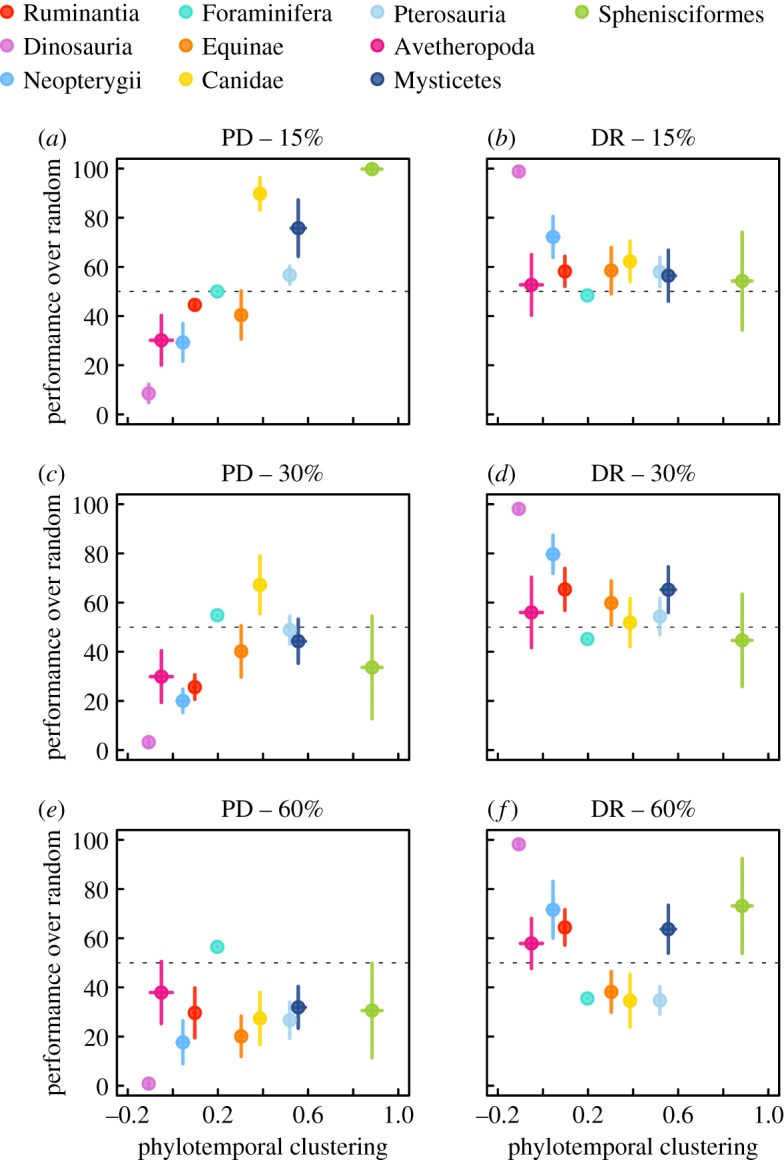
Overall conservation strategy performance. The percentage of time slices where prioritizing diversity, or PD-maximizing (*a*,*c*,*e*), and speciation, or DR-maximizing (*b*,*d*,*f*) strategies perform better than random conservation, plotted against phylo-temporal clustering, which reflects whether synchronous divergence and extinction events are also phylogenetically clustered. Each row represents performance under a different proportion of conserved lineages (from top to bottom: 15, 30, 60%). Coloured points reflect averaged metrics, and bars show ±s.d. across 100 trees (except for Foraminifera, for which we analyse one tree). The horizontal dashed line represents 50% performance: below this line, a given strategy performs worse than random conservation on average. (Online version in colour.)

Figure 3 presents the performance through time of the two prioritization strategies across each of the 10 trees at 30% conservation (electronic supplementary material, figures S6 and S7 also present 15 and 60%); figure 4 presents the overall relative performance across all three conservation intensities. We highlight three major patterns. First, while the two conservation strategies are not directly contrasting, their performance is generally negatively correlated. Second, sets created to maximize PD consistently led to lower total biodiversity over the long term than did sets of random lineages (figure 4*a,c*,*e*). Conversely, the speciation-rate-maximizing conservation strategy performed as well or even slightly better than random lineage selection (figure 4*b*,*d*,*f*). Third, there is marked variation among trees both in the relative performance of the schemes and the effect of conservation intensity. Though our sample size is small, several correlated measures of overall tree shape are good predictors of the performance of the PD-maximizing strategy, especially when fewer lineages are conserved. Figure 4 presents the results for one measure, phylo-temporal clustering, which captures the extent to which members of entire clades either diversify in concert or are quiescent. We consider other measures in electronic supplementary material, figure S4 and table S1, and find increased performance of the PD-maximizing strategy associated with the smaller trees and with the trees with relatively short internal branches in our dataset.

Our main result—that if we conserve lineages that maximize PD, we generally get less subsequent biodiversity across our trees—is troubling. The logic underpinning why we should prioritize evolutionary diversity is best described by the sampling effect: given the vagaries of future diversification, choosing distantly related lineages in the present increases the chances of sampling some that will subsequently diversify. The logic is also robust—maximizing PD is achieved by choosing lineages from each sister clade at each split as one moves from the root to the tips [28]. On reasonable trees, such a PD-maximizing strategy is likely to capture some tips from clades that have diversified in the recent past as well as some that are part of early-diverging lineages; at the limit on a perfectly balanced tree, PD chooses tips non-randomly, but all chosen species have the same DR.

However, the poor performance of the PD-maximizing scheme coupled with the better performance of the speciation-rate-maximizing scheme suggests that sampling species-poor lineages is actually a bad bet: slow diversifiers appear to remain small [21,28], while lineages that are currently speciating are more likely to grow into the macroevolutionary future. It is well known that the shape of phylogenies of extant species is consistent with variation in DRs among entire subclades [29], and so this interpretation for our results extends this latter mechanism to palaeo-phylogenies more generally.

As we increase sampling, the sets of conserved species under the three strategies will tend to be more similar because sampled sets will include more overlapping sets of taxa, and the performance of all conservation strategies should converge on the performance of random samples, as they do (figure 4*e*,*f*). The interpretation of the relative performance at different conservation intensities depends on how severe one thinks future extinction is going to be. Regardless, there is no indication that milder anthropogenic extinction would support either conservation strategy: the performance of the speciation rate-maximizing strategy also deteriorates as sampling increases, suggesting it should not be advocated at this stage.

Considering among-tree variation, it is clear that the performance of each of our conservation algorithms shifts among trees and over time (figure 3; electronic supplementary material, figures S6 and S7). Though several measures of overall tree shape do show promise as predictors of the performance of the two strategies (see electronic supplementary material, figure S4 and table S1), such measures offer little practical use because conservation decisions are made at a single time slice (figure 1)—for us, in the present. We therefore asked the following more practical question: is there information in the tree shape at the time a conservation strategy is implemented that predicts subsequent performance? We can measure tree shape of the reconstructed tree at every time slice (i.e. the tree connecting the extant lineages at that slice) and ask if this shape predicts the performance of either conservation strategy. However, none of our measures (tree balance, γ and two measures of speciation rates) reliably predicted the performance of either conservation scheme across trees (electronic supplementary material, figures S8 and S9).

Generally, our results are consistent with a model where idiosyncratic macroevolutionary events [30] determine the result of conservation algorithms over long time scales. For real trees, random or idiosyncratic behaviour can, of course, be described in retrospect, tree by tree and era by era. The PD-maximizing approach conserves species sitting on long branches and will succeed if such quiescent lineages subsequently diversify. For example, 13 million years ago, a conservationist applying this approach would probably have saved a lineage of American hipparionine horses without any remarkable ecomorphological adaptation [31] that 2 million years later underwent a substantial diversification pulse as it dispersed into Eurasia and Africa. However, none of these Old World hipparionine horses survived after the Pleistocene, highlighting that even the outcome of successful conservation decisions might be fleeting over macroevolutionary time scales. Likewise, a prehistoric conservation biologist focused on fast-diversifying lineages among the ruminants around 24 Ma would have chosen lineages that subsequently evolved into a variety of phenotypes and that made up most of subsequent ungulate diversity. If she had, on the other hand, chosen sets to maximize PD, several mouse deer lineages that subsequently diversified very little would always have been retained, and in this case, random sets would have produced more lineages subsequently.

The patterns we report also speak to what, if anything, defines long-lasting, slow-diversifying lineages, or the long edges in our fossil phylogenies. Limited palaeontological evidence suggests that lineages with long durations in the fossil record might be phenotypically average, and there are arguments that they are generally more prone to persist, and, critically, to produce new forms [20]. In some microbes, generalist taxa tend to predominate diversification dynamics [32]. However, others have argued that while long-lasting lineages have higher climatic tolerances, they show slower speciation and extinction rates [28,33–36]. Our primary result, that the PD-maximizing scheme applied to the fossil phylogenies analysed leads to less subsequent biodiversity than random choice, seems most consistent with the notion that lineages on long branches are statistically unremarkable [17], or even dead ends [21,37]*.* Thus, whether or not such lineages might have average ecomorphologies that help them endure [19], these lineages may not beget future biodiversity [21]. Indeed, in the electronic supplementary material, we document no strong pattern in our trees of persistence for lineages chosen to maximize PD (electronic supplementary material, figure S2).

As with almost all palaeontological data, the trees we have used represent only a subset of their total evolutionary histories (see [38], for example), and so we must assume that their shapes are unbiased with respect to our test. Under simulated random sampling, we find no overall bias, with DR being less sensitive to sampling than PD (electronic supplementary material, figure S3). This could be interpreted as supporting the suggestion that the DR conservation strategy can produce moderate gains in total subsequent biodiversity. Non-random sampling of our palaeo-trees (e.g. due to geographical and temporal patterns of fossil discovery) might also lead to biases [39], and demonstrably have for at least one of the trees included here (all trees include most of the known species of each group, except Mesozoic Avetherapoda [40]). This is an important area for any future work looking at the shapes of palaeo-trees generally.

It is important to highlight that our preferred metric of conservation success (figure 1) (i) is based on the total number of lineages, (ii) integrates this total subsequent diversity following a conservation decision at a particular time slice, and (iii) further sums up this measure across all possible time slices for each group. All three decisions merit comment. The third step, integration over all time slices, should be least controversial—conservation decisions are to be made in the present, and the present time slice is likely to be arbitrary with reference to the past history of any particular clade of interest, meaning we should consider the average effect over many possible time slices.

It is important to highlight that our biodiversity metric only counts lineages, and does not, perforce, include any concept of disparity, making it an incomplete measure of biodiversity. Indeed, the study that helped motivate this work [10] was focused on using PD as a measure of feature diversity, a concept closely aligned with disparity. The study did, however, make the claim that PD may be a strategy to safeguard future diversification as well: ‘We therefore argue that maximizing PD will in turn maximize the options for future diversification … Throughout the history of angiosperms, diversification has been a complex process in which the propensity to diversify was highly labile and dependent upon many different traits at different times’ [10, p. 759]. Initial arguments for safeguarding future diversification potential [11–14,16] were raised as complements to those for preserving present disparity. So, though feature diversity is a more common argument for preserving PD, including it here (measured, e.g. as PD) would lead us to circularly prefer PD-maximizing over speciation rate-maximizing schemes (see Davis *et al*. [41] for a clear discussion of the potentially weak relationship between the accumulation of biodiversity and the accumulation of disparity).

Finally, the time scale for measuring the outcome of conservation decisions on future biodiversity is relevant to arguments about benefits to humans. One could also ask how choices at one time slice affect biodiversity maintenance and production at specific subsequent times (e.g. after 1 or 10 million years), though we know of no defensible way to choose such a window. More generally, there is an active debate on the benefits of conserving the means of production of future biodiversity over short time scales (see [7,9,42]). Conservation decisions flow from a complex interacting nexus of social, economic and scientific concerns. Policy makers and managers generally need to balance competing priorities and provide meaningful evidence of improvement across these concerns on time scales that are meaningful to society. Social and economic factors, and the politics arising from these, are always immediate, and conservation justification relying on expected events over millions of years will rarely gain traction [43]. But if our palaeo-phylogenies are representative, the argument that maximizing current PD safeguards future lineage production [10,11] is empirically unsupported in any case. If anything, we might want to reconsider approaches that explicitly safeguard ongoing diversification [11] instead of strategies that maximize current disparity. Most likely, though, the macroevolutionary future cannot be predicted based on tree shape alone, and we should consider other biodiversity benefits to help us prioritize species and areas for conservation activities. Given the urgent need for operational and scalable triage approaches, we call for more directed tests of the benefits to humans arising from the approach that aims to maximize standing PD.

## 3. Material and methods

### (a) Phylogenetic data

Our dataset is made up of 10 empirical fossil phylogenetic datasets, selected via an extensive search of the relevant literature. In order to qualify, the tree needed to be resolved to the species level, and to contain extensive fossil information where phylogenetic affinities of fossil taxa are reasonably well established and where the known stratigraphic range is included as part of the length of the tips because our method relies on future diversity trajectories (figure 1; see the electronic supplementary material). An overview of the dataset is presented in figure 2 and further details are provided in the electronic supplementary material.

### (b) Evaluation of conservation strategies

We used two common measures of evolutionary diversity to guide our sampling of species for conservation. The first is Faith's PD [8], which is simply the length of the minimum-spanning tree connecting a set of species to the root of the embedding tree. A greedy algorithm [44] was used to identify (non-unique) sets of species guaranteed to maximize PD for a given set size. The second measure is the species-level lineage DR, the inverse of one measure of evolutionary isolation [24]. DR is higher for species that have many close extant relatives, reflecting ongoing high rates of speciation. By contrast, species with fewer close relatives generally contribute more to the PD of a set and such species have lower DR.

A schematic workflow of our procedure is depicted in figure 1. The core logic of our approach is to model conservation decisions made at geological time slices in the past, whereby 15, 30 and 60% of species are selected for conservation and all others are pruned from the tree to simulate anthropogenic extinction. At each such time slice, we conserve the same number of lineages that maximize PD, that maximize DR and at random, and then compare subsequent diversity.

Because we have many trees and many possible sets of species that fulfil each conservation strategy, our sampling proceeded as follows. Given a tree and a time slice, we produced 100 pruned trees that each conformed to a particular conservation strategy (e.g. 100 different PD-maximizing trees under 30% sampling). We then produced a median lineages-through-time plot for these 100 pruned trees, using the package *palaeotree* [45], and took the area under that the median plot as our measure of subsequent diversity. Our relative measure of performance at a time slice is this median area over the corresponding area under the median lineages-through-time plot generated from 100 trees randomly pruned at that time slice, a ratio that we log-transformed. Thus, slices where PD- or DR-maximizing strategies perform better than random will yield a value above 0 and slices where random conservation performs better will yield a value below 0. We then repeated this at every 1-million-year time slice (every 5 million years for the very deep dinosaur, pterosaur, Avetheropoda and Neopterygii trees, which produced similar total numbers of time slices as the others), discarding slices with fewer than five lineages present, and recorded the percentage of time slices where our strategy did better than random. This is our performance metric for a sampled tree. We then repeated this for each of 100 trees from our posterior sample of trees per taxon (except for Foraminifera, where we have only one tree), and report the mean percentage across trees (e.g. figure 4).

### (c) Tree shape metrics

We estimate two sets of tree shape measurements. The first set of metrics considers whether the overall performance of PD- and DR-maximizing strategies can be predicted by overall tree shape. Here, we measured overall tree balance (*β*), the proportion of the total length of the tree belonging to the tips (what we call ‘fossil gamma’, *γ*_f_) and ‘phylo-temporal’ clustering (abbreviated PTC). The phylo-temporal clustering measure is the extent to which temporally synchronous divergence and extinction events are also phylogenetically clustered (see electronic supplementary material, figure S1). A clade where different subclades successively replace each other in replicated radiations through time (e.g. in canids [46]) will have a higher phylo-temporal clustering score than clades where all subclades radiate (or go extinct) at the same time or where events are widely scattered. We hypothesized that a pattern of clade replacement would best predict when the diversity-maximizing conservation strategy would yield biodiversity dividends. More methodological details are provided in the electronic supplementary material.

The second set of metrics was measured on the reconstructed phylogeny at each time slice—the tree connecting those lineages extant at that slice—in order to determine whether tree shape on extant species might predict PD- and DR-maximizing performance. We compute tree balance (*β*), the distribution of splitting times through the tree (*γ*), median DR (are lineages at that slice diversifying at high or low rates) and DR skewness (whether fast- or slow-diversifying lineages are more prevalent in the reconstructed tree).

## Supplementary Material

Cantalapiedra_tables_figures_ESM.pdf

## Supplementary Material

Dataset S1

## Supplementary Material

Dataset S2

## Supplementary Material

Dataset S3

## Supplementary Material

Dataset S4

## Supplementary Material

Dataset S5

## Supplementary Material

Dataset S6

## Supplementary Material

Dataset S7

## Supplementary Material

Dataset S8

## Supplementary Material

Dataset S9

## Supplementary Material

Dataset S10

## Data accessibility

All phylogenetic datasets analysed are available from the Dryad Digital Repository: https://doi.org/10.5061/dryad.gd8038s [47].
