# Supplementary material for: Conserving evolutionary history does not result in greater diversity over geological time scales
Source: Proc Biol Sci. 2019 Jun 5;286(1904):20182896. doi: 10.1098/rspb.2018.2896 (PMC6571466; doi:10.1098/rspb.2018.2896)
Supplement: Cantalapiedra_tables_figures_ESM.pdf [file rspb20182896supp1.pdf]

Electronic Supplementary Material for:

Conserving evolutionary history does not result in greater diversity over geological timescales

Cantalapiedra JL, Aze T, Cadotte M, Dalla Riva GV, Huang D, Mazel F, Pennell MW, Ríos M & Mooers AØ

This PDF file includes:

Supplementary Text
Figs. S1 to S9
Table S1
Caption for Databases S1 to S9
References for SM reference citations

Other supplementary materials for this manuscript include the following:

Datasets S1 to S10

Supplementary Text

Material and Methods

Fossil Phylogenies

Our approach to testing net biodiversity production following hypothetical conservation prioritization requires well resolved phylogenies that capture both speciation and extinction through time. We were able to identify ten empirical fossil groups that had reasonably sampled and tip-dated phylogenies (or posterior sets of phylogenies) resolved to the species level. For nine of these groups, we used the published tree(s) or recreated distributions of trees using the data and platforms from the original publication. We generated the first tree ourselves. The trees are described as follows (ordered by decreasing number of tips, as in Fig. 1):

1. A new phylogeny of ruminants that includes 1,277 living and fossil species. See methods below.

2. A distribution of Mesozoic dinosaur metatrees (n = 962) , where no taxa survive the K/Pg boundary (66 Ma), based on source trees generated from morphological characters aimed at

maximising OTU's and filtered via a taxonomic reconciliation approach, with a root depth of 181 my (root age estimated at ~250 Ma) [1].

3. A tree of Permian–Early Cretaceous crown Neopterygii (n = 617) [2]. A posterior distribution of 100 trees was obtained directly from the published supplementary information. These trees were dated fitting the tree topology to temporal stratigraphic ranges. Root depth around 240 myr (root age estimated at ~340 Ma).

4. A precisely calibrated macroperforate planktonic foraminifera tree (n = 339) based on morphological data of fossil populations exclusive of molecular data [3], with a root depth of 70 myr.

5. A recently published tree of Neogene horses (n = 138) obtained using an informal supertree approach and calibrated using stratigraphic ranges [4], with a root depth of 18 my.

6. A canids synthetic phylogeny based on morphological data (n = 140) inferred using a tip-dating approach [5], with a root depth of 39 my.

7. A tree of pterosaurs (n = 109) [6], with a total depth of 183 myr (root divergence time around 250 Ma). We generated a tree distribution using the *paleotree* package (dating based on stratigraphic ranges) using the original R code from Benson et al..

8. A tree of Mesozoic Avetheropoda (n = 89) [7]. We used a posterior distribution of 100 trees from the *MrBayes* run (tip-dating) that accounts for sampled ancestors (SA). Tree file available from the supplementary information of the original paper.

9. A tree of Mysticetes (n = 78) [8]. We generated a posterior distribution by running the BEAST2 xml file provided in the supplementary information of the publication, and sampled 100 trees from that for our analyses. Root depth is centred on 44 Ma.

10. A tree of penguins (n = 55) [9]. Trees were obtained using a tip-dating approach by running the BEAST2 xml file provided in the supplementary information of the publication. We run the analysis with a relaxed clock and that allowed for sampled ancestors (SA). Root age around 67 Ma.

The Ruminantia tree. Ruminants have a rich fossil record spanning the last 45 myr [10]. The taxonomic affinities of extinct and living ruminants are relatively well known (especially for a group so diverse) thanks to extensive taxonomic research and a constantly-growing phylogenetic evidence [11]. We first reviewed and updated a recently published taxonomic list of fossil ruminants [10]. The final list includes 1069 extinct ruminant species. We then used the extensive taxonomic literature for ruminants [12-90] to built a 'taxonomic table'. In this 'taxonomic table' species were placed in rows, and different taxonomic levels were placed as columns (e.g. crown ruminantia, stem Pecora, crown Pecora, family, tribe, etc). In

total, 17 columns were used, including, for example, ‘among tribes’ (and other similar levels), for fossils whose position among two or three tribes is not known. For example, the steppe bison (*Bison priscus*), would show Bovidomorpha, stem_Bovidae, Bovidae, stem_Bovinae, Bovinae, Bovini in different columns.

Using mitochondrial DNA sequences for 137 ruminant species from Hassanin et al. [91], we built a MrBayes-ready nexus file into which taxonomic information was added using partial constraints (or ‘backbone’ constraints). Backbone constraints force a defined set of taxa to form a monophyletic clade, and exclude a second defined set of taxa from that monophyletic clade. Taxa that are not specified in either of the two sets are free to move within and outside the defined clade. We created an R script that translated the ‘taxonomic table’ into such constraints, so that taxa that had been placed into a tribe, for example, would be forced to be placed in that tribe but excluded from other tribe-level constraints. A taxon with information only at the family level would not be placed in any particular tribe, and thus would be free to move within the assigned family. A similar approach was used for placing living species up to the family level (including also the 71 living species without molecular information). A total of 91 partial constraints were used above the genus level. Genera were assumed to be monophyletic, using 206 hard constraints. Ages of fossil tips were sampled from the temporal range assigned to the oldest occurrence of each species. The final dataset includes 1277 species (208 extant, 1069 extinct) We used the fossilised birth-death process (FBD) and a IGR relaxed clock, setting variance on clocks to MrBayes default. We defined a uniform age prior the node containing Pecora, with a lower limit equal to the upper limit of the oldest occurrence of a pécora (22.8 Ma) and the upper limit in 25 Ma. We set up two independent runs, with four chains each, for 40 million generations, sampling every 10,000, and discarding the first 30% as burnin. 100 trees were retained from the resulting posterior sampled tree distribution for further analyses. Based on occurrence data, we extended the tips of the resulting tree to include the stratigraphic ranges, assigning random ages from the oldest occurrence to each tip.

Measures of Evolutionary Diversity

We used two common measures of evolutionary diversity. The first is Faith’s phylogenetic diversity (PD) [92], which is the length of the minimum-spanning tree connecting a set of species to the root of a tree. A greedy algorithm [93] was used to identify (non-unique) sets of species that is guaranteed to maximise this quantity. The second measure is species-level lineage diversification rate (DR; see ref. [94]), which is the inverse of the simple equal splits measure of evolutionary isolation [95]. DR is higher for species at the end of short branches, reflecting fast-diversifying lineages and close relative. In contrast, species with fewer close

relatives are found at the end of longer branches, and generally contribute more to the PD of a set. Such species have lower DR values. Extant sister species have the same DR scores.

Evaluating PD and DR efficacy through time

The core logic of our approach (outlined in Fig. 2) is to model conservation decisions made at geological time slices in the past, whereby 15, 30, and 60% of species are selected for conservation while all others are pruned from the tree, mimicking anthropogenic extinction. Because neither the PD maximizing, DR maximizing, nor the random sets are unique, we repeated our sampling for each time slice 100 times. Median LTT areas were then computed for the 100 resulting LTT trajectories at each slice (and for each tree) using the package *paleotree* [96] and stored. For each slice and tree, we compare the resulting LTT area from a strategy where PD (or DR) is maximized with the LTT area that would result if the same number of random species had been saved at that time slice (Fig. 2). To do so, we compute the $\log_{10}$ of the ratio of the LTT area from our PD (or DR) strategy and the LTT area obtained from random conservation. Thus, slices where maximizing PD (or DR) performs better than random will yield a value above 0. At slices where random conservation performs better, we will get a value below 0.

We repeat this at one million year intervals, but evaluate performance only for time slices with at least 5 lineages extant at a time slice. For very deep trees (dinosaurs, pterosaurs, Mesozoic birds and Neopterygii) we considered time slices every 5 myr to obtain a comparable number of observations.

Our overall comparable metric of performance for a tree is the proportion of slices where PD- (or DR-) based strategy performed better than random i.e. the number of slices in each tree where the LTT area ratio was above 0 divided by the total number of slices in the tree. We then take the average of this quantity across 100 trees from the posterior distribution for each taxon (except for foraminifera, where we have only one tree).

Lineage persistence under different conservation strategies

Conserving taxa that maximize PD generally means choosing tips from longer-than-average branches (from the tip to the root). There is an argument that "older" lineages may have lower than average persistence into the future [97], a mechanism that could contribute to the poor performance of the PD-maximising strategy. However, contrasting theory proposes that under constant physical settings, extinction risk may be lineage-age independent [98, 99], and that furthermore during major extinction events, it is high-turnover (short branch-length) taxa that suffer most [100, 101]. The strategy that maximizes PD bets on the possibility that tips emanating from longer branches would serve as the origin of new diversity, for example

as new ecological opportunities open up [101]. Such macroevolutionary opportunities are often the result of environmental change [102]. Ancestrally slow-turnover lineages may themselves disappear after giving rise to such new designs if the ancestral adaptive zone disappears as a result of such change even as the clade itself persists. Of course, it may be that the new adaptive zone is also ephemeral such that the clade is also short-lived. In an attempt to test these possibilities, we compared the subsequent persistence of lineages identified as emanating from long or short branches. For each clade, tree, and time slice, we estimated the tip-specific diversification rate (DR) [94] of the currently extant species. We grouped tips below and above the 50% quantile of their DR scores (tips emanating from long vs. short branches, respectively), and measured the subsequent persistence of the clade (i.e. the time till the entire clade has gone extinct, or the present, whichever is longer) started at that tip, standardized by the temporal depth of the slice. We then compute Welch T-tests on the average persistence of the two types of tips. Results, plotted as the difference in the two means at each time slice, are shown in Fig. S2. Overall, we find no clear pattern: lineages emanating from both short and long branches seem to generate resilient lineages. We note that other, more exact tests of these paleontological predictions using fossil trees such as ours are possible and desirable.

Sampling effects on the shape of fossil phylogenies

Despite including extensive fossil tips, most of our trees are very sparse, and incompleteness could have impacts in our PD-maximizing and DR- maximizing implementations, especially if they lead to bias. To test for the robustness of our approach in different scenarios of paleontological sampling, we conducted a set of simulations. We built 500 birth-death trees with approximately 1000 total tips, using a speciation rate (λ) of 1 and an extinction rate (μ) of 0.5. We estimated DR for all of the extant tips across the tree distribution, and ranked them according to this DR score. We then randomly dropped 90% of the extant tips in all the trees and repeated the same procedure. This time, for each tree, we used the 50% quantile of the DR scores to classified tips as high DR (i.e. those we would choose using the DR metric) or low DR (i.e. those that we would choose for the max PD set). We then tested for a correlation (Pearson) in the DR-based ranks of the tips in the complete and the subsampled trees, for all the surviving tips, and for the high-DR tips and for the low-DR tips separately.

Our results show that, if sampling is random across lineages, and even when we just recover a 10% of the species, the overall shape of the tree as captured by DR, is retained ($r = 0.329$; Fig. S3). In particular we found that high-DR tips carry a stronger signal of the underlying tree ($r = 0.274$) than do low-DR tips ($r = 0.102$).

Predictive tree shape metrics

Tree-based conservation approaches will rely on reconstructed phylogenies (phylogenetic trees of living species). To test whether typical tree-shape metrics applied to living species phylogenies can predict future performance of PD and DR-based conservation strategies, we extracted the reconstructed phylogeny based on the currently extant lineages at each time slice, and measured tree balance (using β [103]), the distribution of splitting times across the tree (with γ [104]), median DR (the average rate of speciation at that time slice), and DR skewness (a measure of the variation among lineages in speciation). For each clade, we calculated Pearson's correlations between performance of PD and DR maximizing strategies and each of these metrics, combining all sampled trees and all time slices together (Fig S9).

Overall tree shape metrics

The performance of our PD and DR strategies might be expected to be related to the shape of the entire tree (as depicted in Fig. 2). We computed three metrics we considered a priori to be potential predictors and compared each with overall performance of the strategies. The first, tree balance (β) captures the autocorrelation of speciation events along lineages (ie lineages with high speciation rates remain high, and lineages with low speciation rates remain low), and was expected to correlate positively with the performance of DR and negatively with the performance of PD. The second is the proportion of the total length of the tree belonging to the tips (what we call fossil gamma, γ_f). This will be low if speciation and extinction events are correlated along lineages, such that many tips go extinct while short, and DR might not perform well in such situations. The third is a conceptually new measure of phylo-temporal clustering, and captures the degree of to which iterative speciation and extinction events occur within clades at the same time. A clade where different subclades successively replace each other in replicated radiations (e.g. in canids; see ref. [105]) will have a higher phylo-temporal clustering than a phylogeny in which all subclades radiate (or go extinct) in concert or at random. A PD maximizing strategy might be expected to do well under such replacement dynamics. To estimate the phylo-temporal clustering we first computed two distance matrices from a given tree (see Fig. S1). The first matrix contains the phylogenetic distances between the vertices in the tree, and was calculated using the *dist.nodes* function in the *ape* library [106]. The second matrix contains the distances in time between the vertices in the tree, such that contemporary vertices are separted by shorter distances. These pair-wise vertex distances where estimated using internal code in the *geiger* library [107]. In a scenario of iterative radiation, diversification events that are closer

in the tree (i.e. in a subclade) should be also closer in time than events in other parts of the tree, and thus we expect a stronger —positive— correlation between the two matrices in a Mantel test. Our 'phylo-temporal' clustering metric is the correlation coefficient between the two sets of distances. We report the median correlation coefficient across trees.

Each of these measures does predict the performance of the conservation strategies (Table S1, Fig. S4). However, all three are inter-correlated across our sample of trees (Fig. S5), meaning we cannot disentangle mechanism.

Supplementary Figures

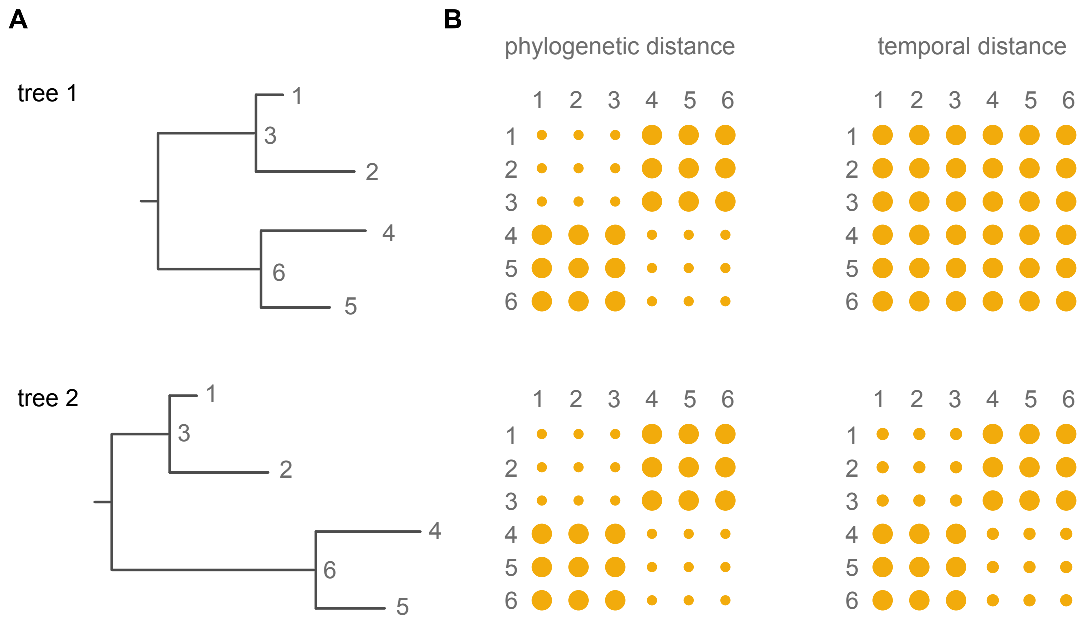

Fig. S1. Schematic representation of the estimate of phylo-temporal clustering (PTC) for two trees (A). For each tree we computed two distance matrices (B). The first matrix contains the phylogenetic distances between the vertices (so, nodes and tips) in the tree (larger dots represent larger phylogenetic distances). The second matrix contains the distances in time between the vertices in the tree, such that cotemporary vertices show shorter distances (here represented by smaller dots). Our phylo-temporal clustering metric is the correlation coefficient between the two distance matrices using a Mantel test. In tree 2 vertices that are close on the tree are also more contemporary, leading to a higher correlation between matrices and a higher PTC. In the figure the root-node is not included in the matrices for the sake of simplicity.

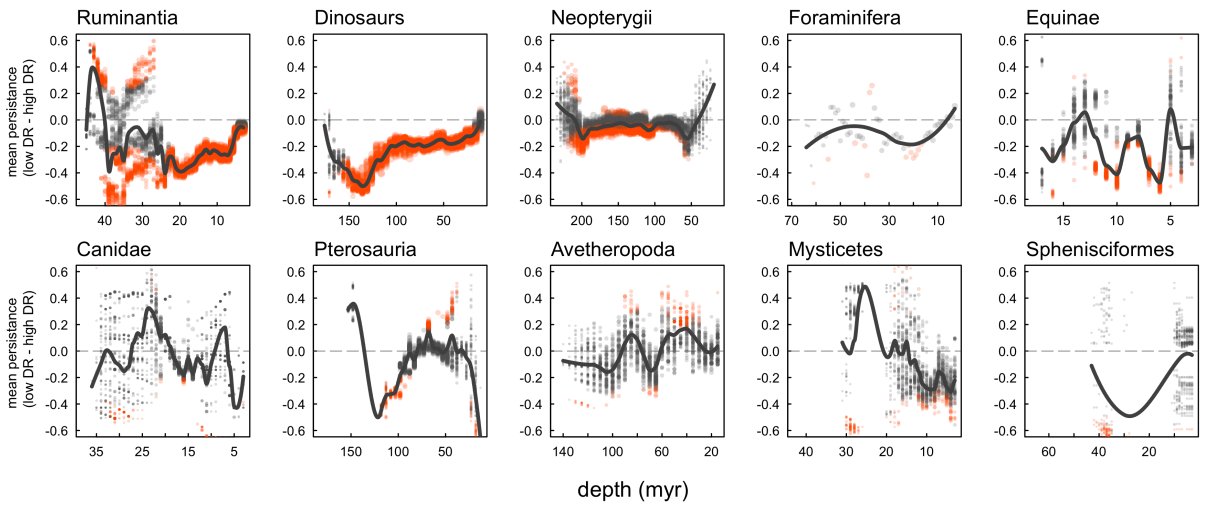

Fig. S2. Relative future persistence in lineages descending from low DR tips and high DR tips. The difference between the means of relative future lineage duration in low DR and high DR tips as measured at each time slice is plotted through time. Red points reflect nominally significant ($p<0.05$) differences according to Welch T-tests. Point sizes are proportional to the number of tips found at each time slice. LOESS curves were fitted to show temporal patterns. An optimal smoothing parameter for the LOESS fit was selected using an Akaike Information Criterion (AIC) [108] so that the resulting curve captures the general trend, and reduces influence of extreme points. Panels are presented in the same order as in Fig. 2.

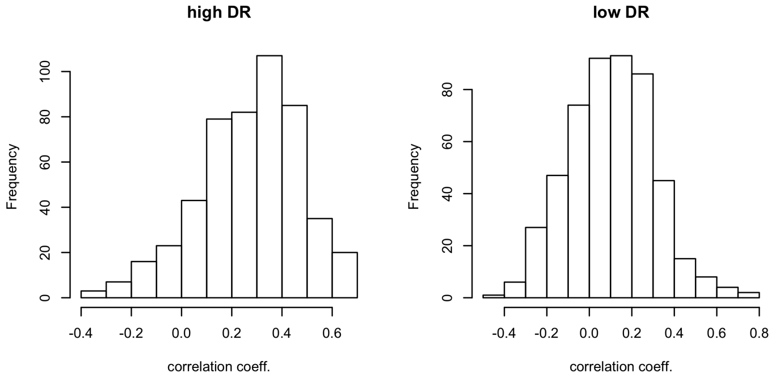

Fig. S3. Correlation of ranked DR scores between 500 complete and subsampled (10%) simulated birth-death ((n~1000 tips, λ =1.0, μ =0.5) trees. High DR tips would be chosen under the speciation-rate strategy, and low DR tips would include many tips chosen under the PD-maximizing scheme.

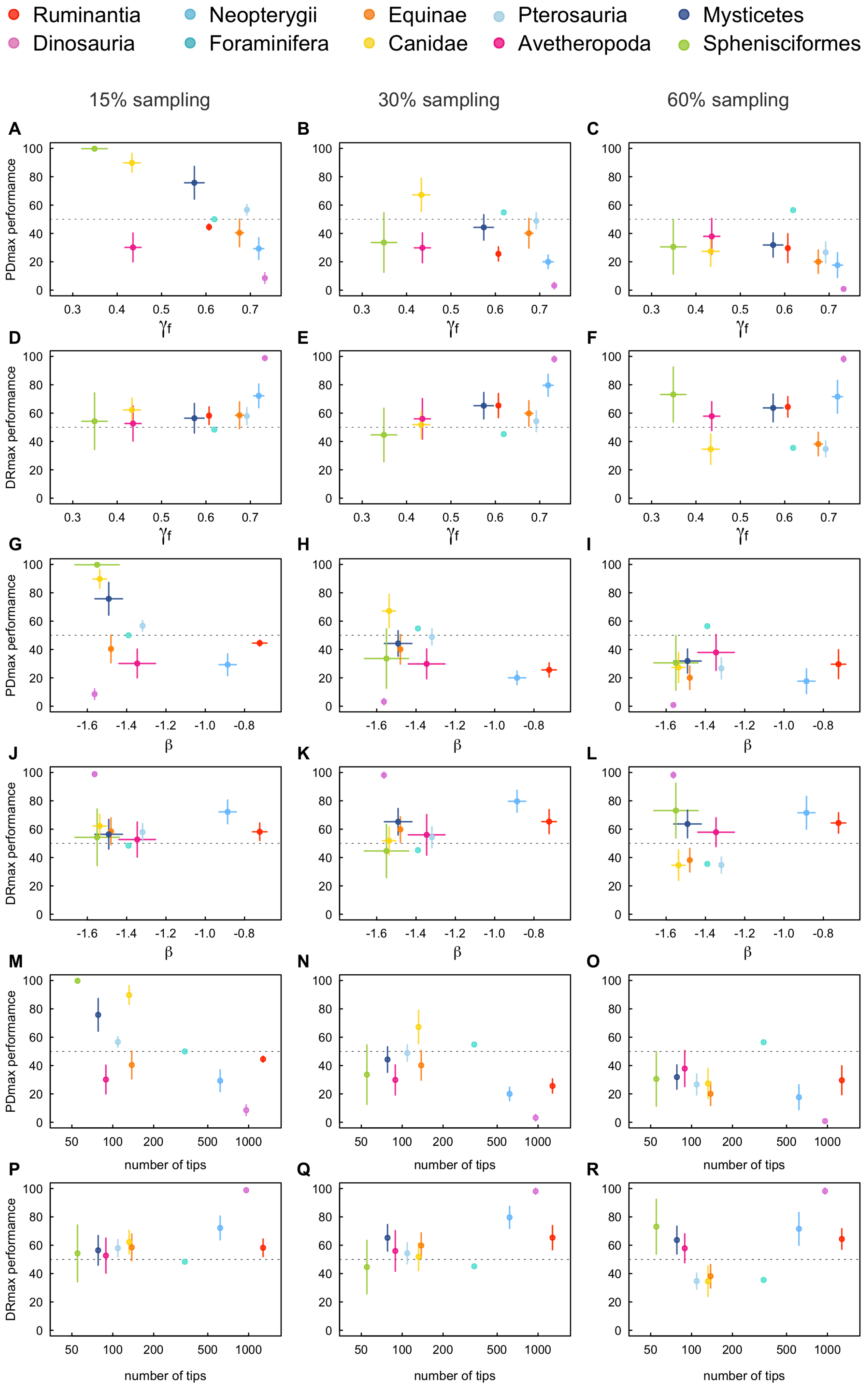
Ruminantia
Dinosauria
Neopterygii
Foraminifera
Equinae
Canidae
Pterosauria
Avetheropoda
Mysticetes
Sphenisciformes
15% sampling
30% sampling
60% sampling
A
B
C
D
E
F
G
H
I
J
K
L
M
N
O
P
Q
R
PDmax performamce
DRmax performamce
γ_f
β
number of tips

Fig. S4. Performance of PD-maximising and DR-maximising conservation strategies, measured as the percentage of time slices where such strategies perform better than random conservation, plotted against the full tree measures: fossil gamma (γ_f, the proportion of tree length held by the tips), tree balance (β), and the number of tips. Each column represents performance under a different proportion of conserved lineages (from left to right 15%, 30% 60%). Coloured points reflect averaged metrics across 100 trees (except for foraminifera, which is a single tree). Dashed lines represents the limit below which a given strategy performs worse than random conservation for more than 50% of the time slices. Note the *x* axis for the number of tips is log-scaled.

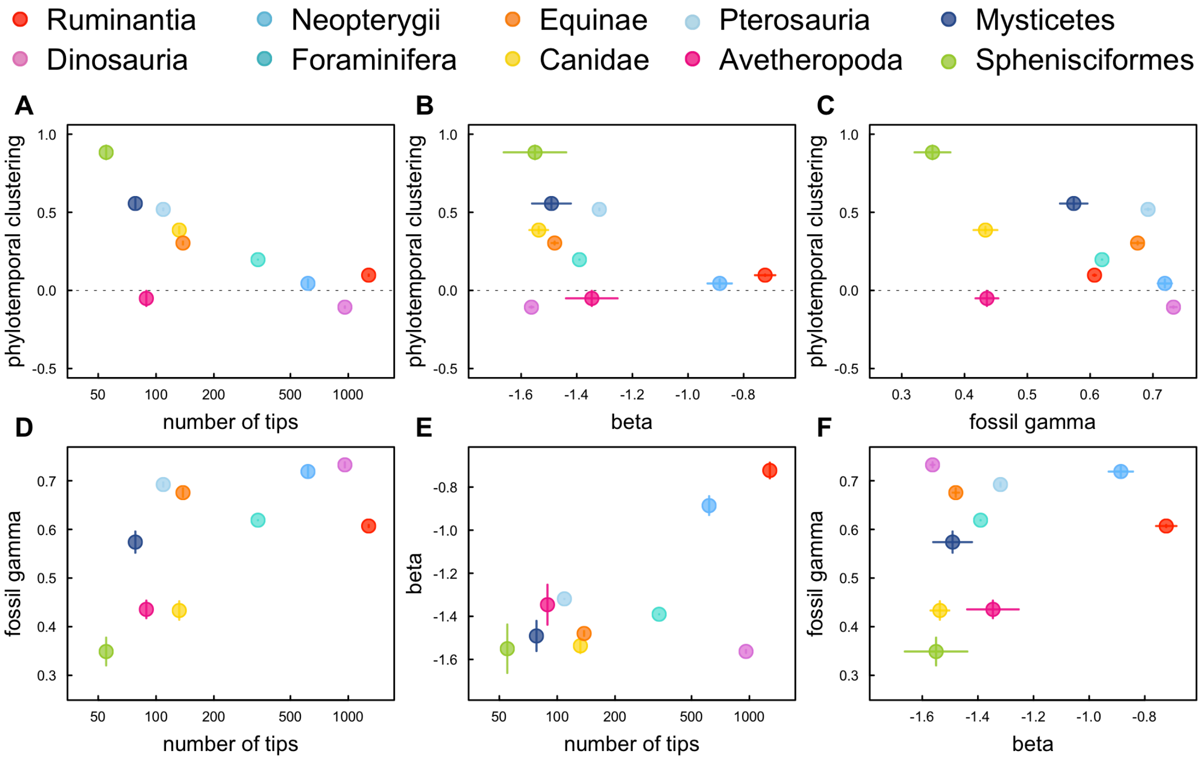

Fig. S5. Phylotemporal clustering, tree size, beta and fossil gamma plotted against each other. Coloured points reflect averaged metrics and bars show ±SD across 100 trees (except for foraminifera, which is a single tree).

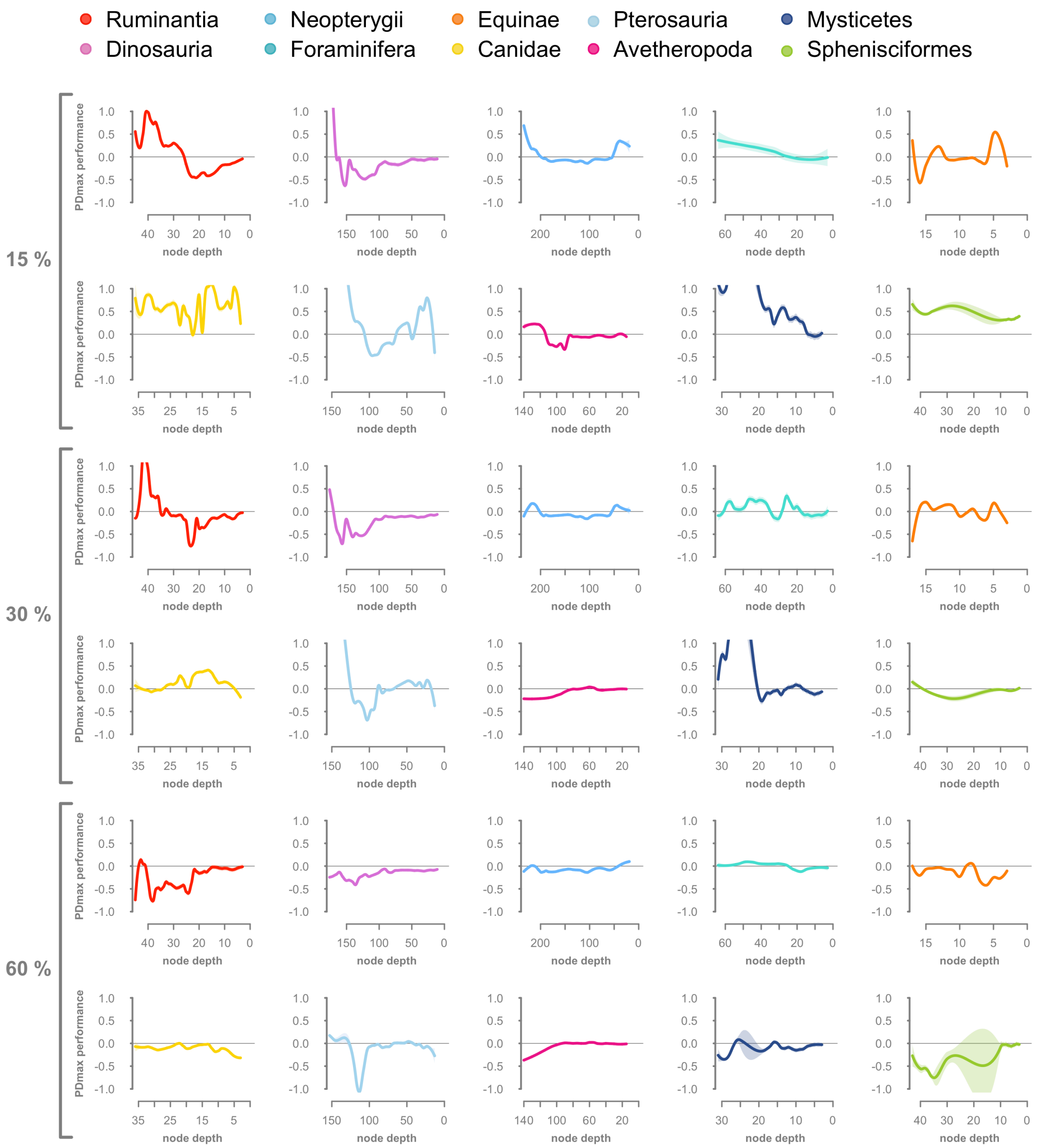

Fig. S6. Performance of the PD-based conservation strategy through time for different proportions of conserved lineages (15%, 30% 60%). We show a LOESS fit to the data over 100 trees (except for foraminifera), where the x values are the times of the time slices in millions of years, and the y values is the $\log_{10}$ of the division of the area under the median LTT plot from our PD-maximising strategy and the area obtained under the median LTT from random conservation. The thin horizontal lines (*y = 0*) represent the value below which the random conservation strategy outperforms the PD maximizing strategy. An optimal smoothing parameter for the LOESS fit was selected using an Akaike Information Criterion (AIC) [108] so that the resulting curve captures the general trend, and reduces influence of extreme points. Shaded areas represent the 95 % confidence interval of the LOESS fit.

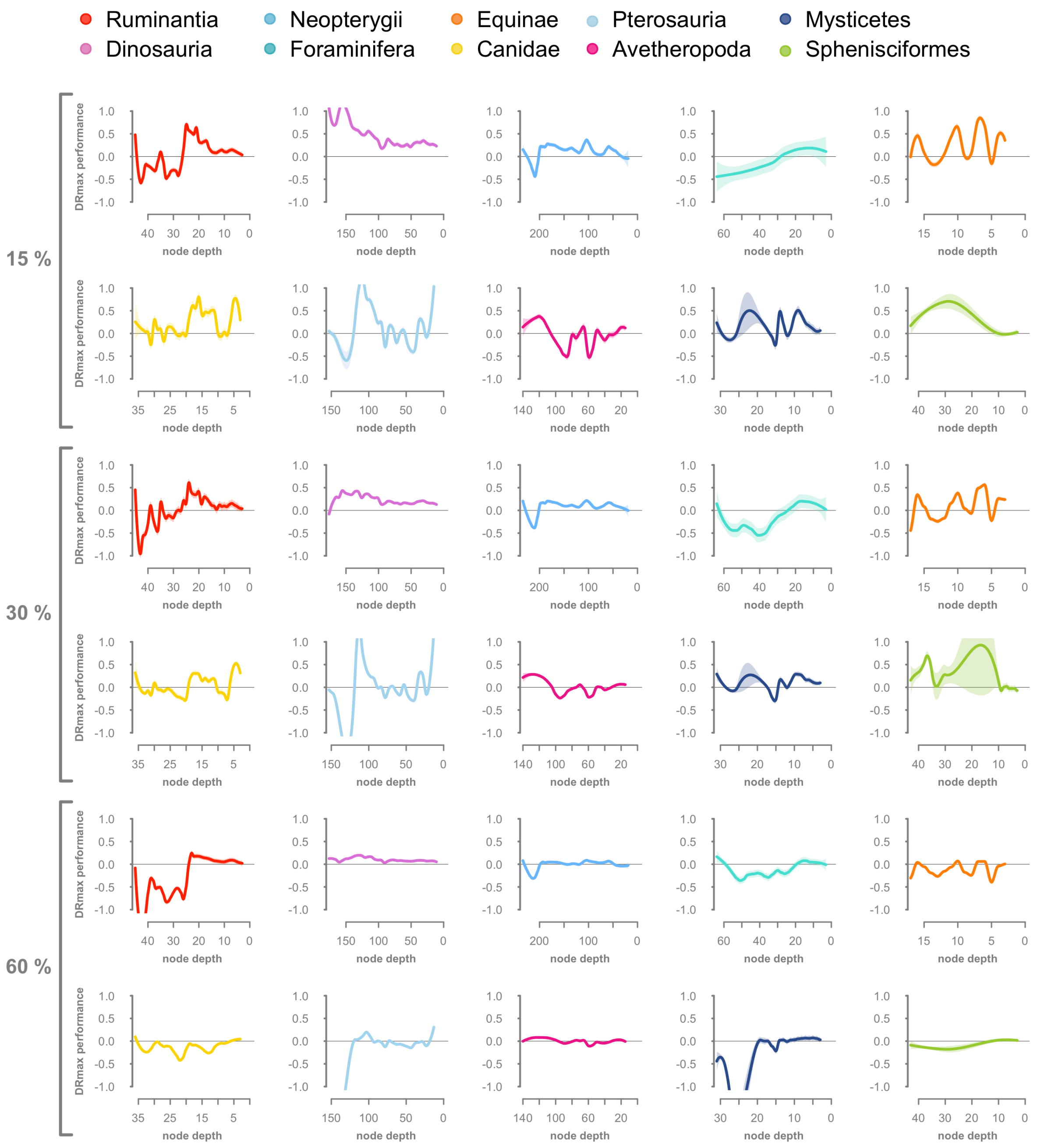

Fig. S7. Performance of the DR-based conservation strategy through time for different proportions of conserved lineages (15%, 30% 60%). We show a LOESS fit to the data over 100 trees (except for foraminifera), where the x values are the times of the time slices in millions of years, and the y values is the $\log_{10}$ of the division of the area under the median LTT plot from our DR maximising strategy and the area obtained under the median LTT from random conservation. The thin horizontal lines (*y* = *0*) represent the value below which the random conservation strategy outperforms the DR maximizing strategy. An optimal smoothing parameter for the LOESS fit was selected using an Akaike Information Criterion (AIC) [108] so that the resulting curve captures the general trend, and reduces influence of extreme points. Shaded areas represent the 95 % confidence interval of the LOESS fit.

tree metric	% lineages conserved	Ruminantia	Dinosauria	Neopterygii	Formainifera	Equinae	Canidae	Pterosauria	Avetheropoda	Mysticetes	Sphenisciformes
β [tree balance]	15%	0.373	0.651	0.140	-0.195	-0.007	0.049	0.130	0.036	0.022	0.155
	30%	0.321	0.239	0.033	0.231	0.280	-0.173	-0.035	0.114	-0.019	0.150
	60%	-0.131	-0.069	-0.054	-0.199	0.078	-0.122	-0.034	0.066	-0.104	-0.024
γ [gamma]	15%	-0.586	0.145	0.321	-0.615	-0.151	-0.033	0.127	0.007	-0.361	0.241
	30%	-0.358	-0.124	0.290	-0.374	-0.084	0.304	-0.058	0.254	-0.258	-0.068
	60%	0.286	-0.284	0.214	-0.437	-0.147	-0.028	-0.373	0.159	-0.075	-0.412
DR median	15%	0.118	0.358	0.282	0.390	-0.052	-0.056	0.308	0.206	-0.043	-0.011
	30%	0.114	-0.230	0.199	0.063	-0.317	-0.071	0.236	-0.598	-0.215	0.052
	60%	0.031	-0.511	0.019	0.202	0.229	0.099	-0.239	-0.574	-0.079	0.036
DR skewness	15%	-0.233	-0.129	-0.353	-0.189	-0.207	-0.050	-0.522	-0.135	-0.319	-0.002
	30%	-0.122	0.343	-0.315	0.070	0.016	0.178	-0.336	0.161	-0.249	0.028
	60%	0.201	0.452	-0.297	0.101	-0.199	0.101	0.149	0.099	0.032	0.071

Fig. S8. Pearson correlation coefficients of diversity-maximizing performance at each time slice with four tree shape metrics of the living species trees at those time slices across 100 trees and for different sampling intensities. Cells are coloured according to the strength of the correlation, with blue and red cells denoting positive and negative correlation respectively. Correlation coefficients above 0.3 and below -0.3 are highlighted with green and red arrows respectively.

tree metric	% lineages conserved	Ruminantia	Dinosauria	Neopterygii	Formainifera	Equinae	Canidae	Pterosauria	Avetheropoda	Mysticetes	Sphenisciformes
β [tree balance]	15%	-0.067	0.144	0.028	0.287	0.041	-0.142	-0.004	-0.017	-0.013	0.021
	30%	-0.058	-0.167	-0.018	0.236	-0.045	-0.113	0.090	-0.032	0.002	0.023
	60%	-0.187	0.049	0.026	-0.048	-0.002	0.130	0.110	-0.032	0.055	-0.098
γ [gamma]	15%	0.389	0.349	-0.199	0.384	0.178	0.213	0.375	0.087	0.072	0.264
	30%	0.274	0.176	-0.225	0.547	0.250	0.204	0.401	0.037	0.142	0.353
	60%	0.505	0.008	-0.222	0.559	0.169	-0.038	-0.043	0.047	0.326	-0.302
DR median	15%	0.103	0.461	-0.157	-0.260	-0.147	-0.034	0.387	0.216	-0.001	-0.028
	30%	-0.012	0.311	-0.263	-0.173	-0.248	-0.032	0.354	0.283	-0.061	0.010
	60%	-0.001	0.269	-0.331	0.172	-0.109	-0.132	-0.357	0.308	0.138	-0.003
DR skewness	15%	0.043	-0.440	0.128	0.045	0.322	0.057	-0.082	-0.038	0.025	-0.034
	30%	0.092	-0.393	0.114	-0.066	0.359	-0.006	-0.175	-0.048	-0.002	-0.056
	60%	0.286	-0.336	0.136	-0.119	0.273	-0.104	0.354	-0.033	0.240	-0.033

Fig. S9. Pearson correlation coefficients of speciation-maximizing performance at each time slice with four tree shape metrics of the living species trees at those time slices across 100 trees and for different sampling intensities. Cells are coloured according to the strength of the correlation, with blue and red cells denoting positive and negative correlation respectively. Correlation coefficients above 0.3 and below -0.3 are highlighted with green and red arrows respectively.

3. Supplementary Table

Table S1. Tree metrics. Species: total number of tips in the tree; slices: the average number of time slices with n>=5 over the sample of 100 trees (except for forams, for which there is only 1 tree); β: beta-measure of tree balance, with the 95% probability distribution of the values across 100 trees (except for forams); γ_f : the proportion of total tree length found in the pendant edges; PTC: the phylo-temporal clustering metric.

	species	slices	β	γ_f	PTC
Ruminants	1277	43	-0.72 (-0.80 — -0.67)	0.61 (0.60 —0.61)	0.099 (0.089 — 0.104)
Dinosaurs	960	33.05	-1.56 (-1.58 — -1.55)	0.73 (0.73 — 0.75)	-0.106 (-0.118 — -0.101)
Perm.-Cret. Neopterygii	617	40.1	-0.89 (-0.96 — -0.80)	0.72 (0.70 — 0.74)	0.047 (-0.007 — 0.089)
Planktonic Foraminifera	339	62	-1.39 (-1.39 — -1.39)	0.62 (0.62 — 0.62)	0.197 (0.197 — 0.197)
Equinae	138	14.94	-1.48 (-1.49 — -1.46)	0.68 (0.66 — 0.69)	0.303 (0.288 — 0.325)
Canidae	132	29.68	-1.54 (-1.59 — -1.48)	0.43 (0.40 —0.47)	0.388 (0.348 — 0.418)
Pterosaurs	109	22.98	-1.32 (-1.32 — -1.32)	0.69 (0.68 — 0.70)	0.519 (0.509 — 0.528)
Mesozoic Avetheropoda	89	23.88	-1.35 (-1.51 — -1.19)	0.43 (0.40 —0.47)	-0.068 (-0.11 — -0.056)
Whales	78	20.69	-1.50 (-1.61 — -1.35)	0.58 (0.54 — 0.61)	0.558 (0.496 — 0.615)
Penguins	55	9.78	-1.55 (-1.68 — -1.34)	0.36 (0.28 — 0.44)	0.913 (0.776 — 0.939)

Datasets S1 to S10. Phylogenetic datasets used in our analyses. Each dataset is a nexus file including a tree distribution with 100 trees (except for planktonic foraminifera).

4. Supplementary References

1. Lloyd GT, Bapst DW, Friedman M, Davis KE. 2016 Probabilistic divergence time estimation without branch lengths: dating the origins of dinosaurs, avian flight and crown birds. *Biol. Lett.* **12**, 20160609-20160604. (doi:10.1098/rsbl.2016.0609).

2. Clarke JT, Lloyd GT, Friedman M. 2016 Little evidence for enhanced phenotypic evolution in early teleosts relative to their living fossil sister group. *Proc. Natl. Acad. Sci. U. S. A.* **113**, 201607237-201607228. (doi:10.1073/pnas.1607237113).

3. Aze T, Ezard THG, Purvis A, Coxall HK, Stewart DRM, Wade BS, Pearson PN. 2011 A phylogeny of Cenozoic macroperforate planktonic foraminifera from fossil data. *Biol. Rev.* **86**, 900-927. (doi:doi:10.1111/j.1469-185X.2011.00178.x).

4. Cantalapiedra JL, Prado JL, Hernández Fernández M, Alberdi MT. 2017 Decoupled ecomorphological evolution and diversification in Neogene-Quaternary horses. *Science* **355**, 627–630. (doi:10.1126/science.aag1772).

5. Matzke NJ, Wright A. 2016 Inferring node dates from tip dates in fossil Canidae: the importance of tree priors. *Biol. Lett.* **12**, 20160328-20160324. (doi:10.1098/rsbl.2016.0328).

6. Benson RBJ, Frigot RA, Goswami A, Andres B, Butler RJ. 2014 Competition and constraint drove Cope's rule in the evolution of giant flying reptiles. *Nat. Commun.* **5**, 3567. (doi:10.1038/ncomms4567).

7. Bapst DW, Wright AM, Matzke NJ, Lloyd GT. 2016 Topology, divergence dates, and macroevolutionary inferences vary between different tip-dating approaches applied to fossil theropods (Dinosauria). *Biol. Lett.* **12**. (doi:10.1098/rsbl.2016.0237).

8. Slater GJ, Goldbogen JA, Pyenson ND. 2017 Independent evolution of baleen whale gigantism linked to Plio-Pleistocene ocean dynamics. *Proc. R. Soc. B* **284**, 20170546-20170548. (doi:10.1098/rspb.2017.0546).

9. Gavryushkina A, Heath TA, Ksepka DT, Stadler T, Welch D, Drummond AJ. 2015 Bayesian total evidence dating reveals the recent crown radiation of penguins. *Syst. Biol.* **syw060**. (doi:doi.org/10.1093/sysbio/syw060).

10. Cantalapiedra JL, Hernández Fernández M, Azanza B, Morales J. 2015 Congruent Phylogenetic and Fossil Signatures Of Mammalian Diversification Dynamics Driven By Tertiary Abiotic Change. *Evolution* **69**, 2941-2953. (doi:10.1111/evo.12787).

11. Bibi F. 2014 Assembling the ruminant tree: combining morphology, molecules, fossils, and extant taxa. *Zitteliana* **B32**, 1-15.

12. Sánchez IM, Cantalapiedra JL, Ríos M, Quiralte V, Morales J. 2015 Systematics and Evolution of the Miocene Three-Horned Palaeomerycid Ruminants (Mammalia, Cetartiodactyla). *PLoS ONE* **10**. (doi:10.1371/journal.pone.0143034).

13. Alexejew AK. 1916 Fauna pozvonochnykh derevni Novo Elizabetovki. (p. 453. Odessa, Odessa.

14. Arambourg C. 1959 Vertébrés continentaux du Miocène supérieur de l'Afrique du Nord *Service de la carte géologique de l'Algérie* **nouvelle série (Paléontologie). Mémoires.** , 1-159.

15. Arambourg C, Piveteau J. 1929 Les Vertébrés du Pontien de Salonique. In *Ann. Paleontol.* (pp. 59-138.

16. Azanza B, Rössner GE, Ortiz-Jaureguizar E. 2013 The early Turolian (late Miocene) Cervidae (Artiodactyla, Mammalia) from the fossil site of Dorn-Dürkheim 1 (Germany) and implications on the origin of crown cervids. *Palaeobiodivers. Palaeoenviron.* **93**, 217-258.

17. Beatty BL. 2010 A new aletomerycine (Artiodactyla, Palaeomerycidae) from the early Miocene of Florida. *J. Vertebr. Paleontol.* **30**, 613-617.

18. Bernor RL. 1978 *The mammalian systematics, biostratigraphy and biochronology of Maragheh and its importance for understanding Late Miocene Hominoid zoogeography and evolution*. Los Angeles, University of California.

19. Bibi F, Bukhsianidze M, Gentry AW, Geraads D, Kostopoulos DS, Vrba ES. 2009 The fossil record and evolution of Bovidae: state of the field. *Paleontol. Electron.* **12**, 1-11.

20. Bohlin B. 1926 Die Familie Giraffidae. *Acta Palaeontol. Sin.* **Peking C 4** 1-170.

21. Bonis L, Bouvrain G, Petculescu A, Stiuca E. 2003 Nouveaux Giraffidae du Miocène supérieur de Macédoine (Grèce). In *Advances in Vertebrate Paleontology "Hen to Panta"* (eds. Petculescu A., Stiuca E.), pp. 5-16. Bucharest, Romanian Academy.

22. Borissiak A. 1914 Mammifères fossiles de Sébastopol. *Mémoires du comité Géologique* **Nouvelle série.** , 2-10.

23. Bover P, Quintana J, Alcover JA. 2010 A new species of *Myotragus* Bate, 1909 (Artiodactyla, Caprinae) from the Early Pliocene of Mallorca (Balearic Islands, western Mediterranean). *Geol. Mag.* **147**, 871-885.

24. Campos PF, Sher A, Mead JI, Tikhonov A, Buckley M, Collins M, Willerslev E, Gilbert MTP. 2010 Clarification of the taxonomic relationship of the extant and extinct ovibovids, *Ovibos*, *Praeovibos*, *Euceratherium* and *Bootherium*. *Quaternary. Sci. Rev.* **29**, 2123-2130.

25. Cooke HBS. 1949 Fossil mammals of the Vaal River deposits. *Memoirs of the Geological Survey of South Africa* **35**, 1-117.

26. Costeur L. 2010 The new Miocene Samos faunas. Book Review. *Acta Palaeontol. Pol.* **55(2)**, 1.

27. Costeur L, Malvesy T. 2010 A "new" *Mesopithecus pentelicus* skull from Pikermi (Late Miocene, Greece). *Hell. Journal. Geosci.* **45**, 45-54.

28. Croitor R, Stefaniak K. 2009 Early Pliocene deer of Central and Eastern European regions and inferred phylogenetic relationships. *Palaeontograph. Abteilung* **287**, 1-39.

29. de Mecquenem R. 1924 Contribution à l'étude des fossiles de Maragha. *Ann. Paleontol.* **13: 135-160; 14: 1-36.**

30. Duranthon F, Escuillié F, Juillard F. 1991 Les vertebres du Miocene Inferieur de Barbotan-Les-Thermes (Gers). *Ann. Paleontol.* **77**, 161-216.

31. Gaudry A, Lartet E. 1856 Résultats des recherches paléontologiques entreprises dans l'Attique sous les auspices de l'Académie. *C. R. Hebd. Seances Acad. Sci.* **43**, 271-274.

32. Gentry A. 2006 A new bovine (Bovidae, Artiodactyla) from the Hadar formation, Ethiopia. *Trans. R. Soc. S. Afr.* **61**, 41-50.

33. Gentry A, Heizmann E. 1996 Miocene Ruminants of the Central and Eastern Tethys and Paratethys. In *The evolution of Western Eurasian Neogene mammal faunas* (ed. Bernor R., Fahlbusch, V. & Mittmann, H.-W.(eds.)), pp. 378-391, Columbia University Press, New York.

34. Geraads D. 2010 Biogeographic relationships of Pliocene and Pleistocene North-western African mammals. *Quat. Int.* **212**, 159-168.

35. Geraads D, Kaya T, Mayda S. 2005 Late Miocene large mammals from Yulafl, Thrace region, Turkey, and their biogeographic implications. *Acta Palaeontol. Pol.* **50**, 523.

36. Ginsburg L, Morales J, Soria D. 2001 Les ruminantia (Artiodactyla, Mammalia) du miocene des bugti (Balouchistan, Pakistan). *Estud. Geol.* **57**, 155-170.

37. Godina AY, Baygusheva VS. 1986 A new species of *Paleotragus* from the upper Pliocenein the sea of Azov Region. *Paleontol. J.* **3**, 68-73.

38. Guan J. 1998 The study on *Shansitherium* from Fugu, Shaanxi. *Mem. Beijing Nat. Hist. Mus.* **56**, 125-131.

39. Hamilton WR. 1973 The lower Miocene ruminants of Gebel Zelten, Libya. *Bull. Br. Mus. (Nat. Hist.) Geol.* **21**, 73-150.

40. Hamilton WR. 1978 Fossil giraffes from the Miocene of Africa and a revision of the phylogeny of the Giraffoidea. *Philos. Trans. R. Soc. Lond. B Biol. Sci.* **283**, 165-229.

41. Harris JM. 1974 Orientation and variability in the ossicones of African Sivatheriinae (Mammalia: Giraffidae). *Ann. S Afr. Mus.* **65**, 189-198.

42. Harris JM. 1976 Pliocene Giraffoidea (Mammalia, Artiodactyla) from the Cape Province. *Ann. S Afr. Mus.* **69**, 325-353.

43. Harris JM, Solounias N, Geraads D. 2010 Giraffoidea. In *Cenozoic Mammals of Africa* (ed. Werdelin L.a.S.W.J.), pp. 805-820. Berkeley Los Angeles London, University of California Press. .

44. Haughton SH. 1921 A note on some fossils from the Vaal River Gravels. *S. Afr. J. Geol.* **24**, 11-16.

45. Hopwood AT. 1934 The new fossil mammals from Olduvai, Tanganyika Territory. *Ann. Mag. Nat. Hist.* **14**, 546-550.

46. Janis CM, Effinger JA, Harrison JA, Honey JG, Kron DG, Lander B, Manning E, Prothero DR, Stevens MS, Stucky RK. 1998 Artiodactyla. *Evolution of Tertiary Mammals of North America* **Volume 1, Terrestrial Carnivores, Ungulates, and Ungulate Like Mammals**, 337.

47. Killgus H. 1922 Unterplioziane Sauger aus China. *Paläontol. Zeitschrift* **5**, 251-253.

48. Klein RG. 1994 The long-horned African buffalo (*Pelorovis antiquus*) is an extinct species. *J. Archaeol. Sci.* **21**, 725-733.

49. Korotkevich YS, Kudryashov B. 1976 Ice sheet drilling by Soviet Antarctic expeditions. In *Ice Core Drilling, Proceedings of the First International Workshop on Ice Core Drilling, Univ. Nebraska* (pp. 63-70. Lincoln, Univ. of Nebraska Press.

50. Kostopoulos DS. 2012 Taxonomic re-assessment and phylogenetic relationships of Miocene homonymously spiral-horned antelopes. *Acta Palaeontol. Pol.* **59**, 9-29.

51. Kostopoulos DS, Athanassiou A. 2005 In the shadow of bovids: suids, cervids and giraffids from the Plio-Pleistocene of Greece. In *Les Ongulés holarctiques du Pliocène et du Pleistocène Quaternaire. Hors-série, 2* (ed. (Ed.) É.C.-B.), pp. 179–190.

52. Kostopoulos DS, Bernor RL. 2011 The Maragheh bovids (Mammalia, Artiodactyla): systematic revision and biostratigraphic-zoogeographic interpretation. *Geodiversitas* **33**, 649-708.

53. Kuehn R, Ludt CJ, Schroeder W, Rottmann O. 2005 Molecular phylogeny of *Megaloceros giganteus*—the giant deer or just a giant red deer? *Zool. Sci.* **22**, 1031-1044.

54. Lister A, Edwards C, Nock D, Bunce M, Van Pijlen I, Bradley D, Thomas M, Barnes I. 2005 The phylogenetic position of the 'giant deer'Megaloceros giganteus. *Nature* **438**, 850-853.

55. Matsumoto H. 1926 On some new fossil Cervicorns from Kazusa and Liukiu. *Scientific Report of Tohoku Imperial University* **Series 2 (Geology) X (2)**, 21-23.

56. Matthew W. 1929 *Critical Observations Upon Siwalik Mammals*, American Museum of Natural History.

57. Melentis I. 1974 *Helladotherium duvernoyi* Gaudry, 1860 aus Pikermi (Griechenland). *Sci. Epetiris.* **14**, 65-73.

58. Petronio C, Krakhmalnaya T, Bellucci L, Di Stefano G. 2007 Remarks on some Eurasian pliocervines: Characteristics, evolution, and relationships with the tribe Cervini. *Geobios* **40**, 113-130.

59. Pilgrim GE. 1911 The Fossil giraffidae of India. *Mem. Geol. Surv. India.* **4**, 1-39.

60. Pilgrim GE. 1912 The vertebrate fauna of the Gaj Series in the Bugti Hills and the Punjab. *Mem. Geol. Surv. India.* **4** 1-83.

61. Pilgrim GE. 1939 The fossil bovidae of India. *Palaeontol. Ind.* **26**, 1-356.

62. Pilgrim GE, Hopwood AT. 1928 *Catalogue of the Pontian" Bovidae" of Europe in the Department of Geology*. London, British Museum (Natural History) Geology; 106 p.

63. Pomel A. 1892 Sur le *Libytherium maurusium*, grand ruminant du terrain Pliocène de l'Algérie. *C. R. Hebd. Seances Acad. Sci.* **115**, 100-102.

64. Qiu Z, Qiu Z, Deng T, Li C, Zhang Z, Wang B, Wang X, Book IN. 2013 Neogene land mammal stages/ages of China In *Fossil mammals of Asia: neogene biostratigraphy and chronology.* (ed. Wang X F.L., Fortelius M), pp. 29-90. New York, NY Columbia University Press.

65. Quinquin M, Mialon O, Isnard V, Massin N, Parinaud J, Delotte J, Bongain A. 2014 In vitro fertilization versus conversion to intrauterine insemination in Bologna-criteria poor responders: how to decide which option? *Fertil. Steril.* **102**, 1596-1601.

66. Ríos M, Sánchez IM, Morales J. 2016 Comparative anatomy, phylogeny, and systematics of the Miocene giraffid *Decennatherium pachecoi* Crusafont, 1952 (Mammalia, Ruminantia, Pecora): state of the art. *J. Vertebr. Paleontol.* **36**, e1187624.

67. Rodler A, Weithofer CA. 1890 Die Wiederkäuer der Fauna von Maragha. *Denkschrift Akademische Wissenschaft, Wien* **57**, 753-572.

68. Rook L, Oms O, Benvenuti MG, Papini M. 2011 Magnetostratigraphy of the Late Miocene Baccinello–Cinigiano basin (Tuscany, Italy) and the age of Oreopithecus bambolii faunal assemblages. *Palaeogeogr. Palaeoclimatol. Palaeoecol.* **305**, 286-294.

69. Schlosser M. 1903 Die fossilen Säugetiere Chinas. *Abhandlungen der Königlichen Bayerische Akademie der Wissenschaften* **2**, 1-22.

70. Schlosser M. 1921 Die Hipparionenfauna von Veles in Mazedonien. *Abhandlungen der (Koniglichen baye-rischen) Akademie der Wissenschaften. Munich.* **29**, 1-55.

71. Sharapov SS. 1974 *Sogdianotherim* - A new genus of the family Giraffidae from the upper Pliocene of Tadzhikistan. *Paleontol. J.* **4**, 86-91.

72. Sickenberg O. 1967 Die Unterpleistozäne fauna von Wolaks (Griech-Mazedonien). I. Eine neue giraffe (*Macedonitherium martini* nov. gen. nov. spec.) aus dem unteren Pleistozän von Griechenland. *Annales Géologiques des Pays Helléniques* **XLV**, 313-336.

73. Singer R, Boné E. 1960 Modern giraffes and the fossil giraffids of Africa. *Ann. S Afr. Mus.* **45**, 375-548.

74. Solounias N. 1981 *The Turolian fauna from the island of Samos, Greece. With special emphasis on the hyaenids and the bovids.* New York, S. Karger; 232 p.

75. Solounias N. 1981 Mammalian fossils of Samos and Pikermi, Part 2. Resurrection of a classic Turolian fauna. *Ann. Carnegie Mus.* **50**, 231-270.

76. Solounias N. 2007 Family Giraffidae. . In *The Evolution of Artiodactyls.The Johns Hopkins University Press, Baltimore, 367 pp.* (ed. In Prothero D.R.F., S.E), pp. 257-277

77. Solounias N, Danowitz M. 2016 The Giraffidae of Maragheh and the identification of a new species of *Honanotherium*. *Palaeobiodivers. Palaeoenviron.* **96**, 489-506.

78. Spassov N, Geraads D. 2004 *Tragoportax* PILGRIM, 1937 and *Miotragocerus* STROMER, 1928 (Mammalia, Bovidae) from the Turolian of Hadjidimovo, Bulgaria, and a revision of the late Miocene Mediterranean Boselaphini. *Geodiversitas* **26**, 339-370.

79. Van der Made J. 2008 New endemic large mammals from the Lower Miocene of Oschiri (Sardinia): observations on evolution in insular environment. *Quat. Int.* **182**, 116-134.

80. van der Made J, Hussain ST. 1994 Horn cores of *Sivoreas* (Bovidae) from the Miocene of Pakistan and utility of their torsion as a taxonomic tool. *Geobios* **27**, 103-111.

81. van Hoepen ECN. 1932 Voorlopige beskryving van Vrystaatse soogdiere. *Paleontologiese Navorsing van die Nasionale Museum, Bloemfontein* **2**, 63-65.

82. Vislobokova I. 2009 A new species of Megacerini (Cervidae, Artiodactyla) from the Late Miocene of Taralyk-Cher, Tuva (Russia), and remarks on the relationships of the group. *Geobios* **42**, 397-410.

83. Vislobokova I. 2011 Historical development and geographical distribution of Giant Deer (Cervidae, Megacerini). *Paleontol. J.* **45**, 674-688.

84. Wagner J. 1861 Nachträge zur Kenntnis der Fossilen Hufthier-Ueberreste von Pikermi. *Berichte der Bayerische Akademie der Wissenschaften* **2**, 78-82.

85. Wagner JA. 1848 Urweltliche Säugthier-Ueberreste aus Griechenland. *Abhandlung der Bayerische Akademie der Wissenschaften* **5**, 333-378.

86. Wang X, Xie G, Dong W. 2009 A new species of crown antlered deer *Stephanocemas* (Artiodactyla, Cervidae) from the middle Miocene of Qaidam Basin, northern Tibetan Plateau, China, and a preliminary evaluation of its phylogeny. *Zool. J. Linn. Soc.* **156**, 680-695.

87. Webb SD. 1991 A cranium of *Navahoceros* and its phylogenetic place among New World Cervidae. In *Ann. Zool. Fenn.* (pp. 401-410, JSTOR.

88. Weithofer A. 1889 Ubre die tertiären Landsäugethiere Italiens. *Jahrbuch d.k.k. Geologische Reichesan* **39**, 55-82.

89. Xue X, Zhang Y, Yue L. 2006 Paleoenvironments indicated by the fossil mammalian assemblages from red clay-loess sequence in the Chinese Loess Plateau since 8.0 Ma BP. *Science in China Series D* **49**, 518-530.

90. Werdelin L, Joseph W. 2010 *Cenozoic mammals of Africa*. Berkeley and Los Angeles, University of California Press; 1008 p.

91. Hassanin A, Delsuc F, Ropiquet A, Hammer C, Jansen van Vuuren B, Matthee C, Ruiz-Garcia M, Catzeflis F, Areskoug V, Nguyen TT, et al. 2012 Pattern and timing of diversification of Cetartiodactyla (Mammalia, Laurasiatheria), as revealed by a comprehensive analysis of mitochondrial genomes. *C. R. Biol.* **335**, 32-50.

92. Faith DP. 1992 Conservation evaluation and phylogenetic diversity. *Biol. Conserv.* **61**, 1-10. (doi:https://doi.org/10.1016/0006-3207(92)91201-3).

93. Bordewich M, Rodrigo AG, Semple C. 2008 Selecting Taxa to Save or Sequence: Desirable Criteria and a Greedy Solution. *Syst. Biol.* **57**, 825-834.

94. Jetz W, Thomas GH, Joy JB, Hartmann K, Mooers AØ. 2012 The global diversity of birds in space and time. *Nature* **491**, 444-448. (doi:10.1038/nature11631).

95. Redding DW, Hartmann K, Mimoto A, Bokal D, Devos M, Mooers AO. 2008 Evolutionarily distinct species capture more phylogenetic diversity than expected. *J. Theor. Biol.* **251**, 606-615. (doi:10.1016/j.jtbi.2007.12.006).

96. Bapst DW. 2012 paleotree: an R package for paleontological and phylogenetic analyses of evolution. *Meth. Ecol. Evol.* **3**, 803-807.

97. Jablonski D. 2002 Survival without recovery after mass extinctions. *Proc. Natl. Acad. Sci. U. S. A.* **99**, 8139-8144. (doi:10.1073/pnas.102163299).

98. Van Valen L. 1973 A new evolutionary law. *Evol. Theory* **1**, 1:30.

99. Raup DM. 1975 Taxonomic survivorship curves and Van Valen's Law. *Paleobiology* **1**, 82-96. (doi:10.1017/s0094837300002220).

100. Vrba ES. 1995 On the connections between paleoclimate and evolution. In *Paleoclimate and Evolution with Emphasis on Human Origins* (eds. Vrba E.S., Denton G.H., Patridge T.C., Burcke L.H.), pp. 24-45. New Haven, Yale University Press.

101. Simpson GG. 1944 *Tempo and mode in evolution*. New York, Columbia University Press.

102. Barnosky AD. 2001 Distinguishing the effects of the Red Queen and Court Jester on Miocene mammal evolution in the northern Rocky Mountains. *J. Vertebr. Paleontol.* **21**, 172-185.

103. Aldous DJ. 2001 Stochastic Models and Descriptive Statistics for Phylogenetic Trees, from Yule to Today. *Statist. Sci.* **16**, 23–34.

104. Pybus OG, Harvey PH. 2000 Testing macro–evolutionary models using incomplete molecular phylogenies. *Proc. R. Soc. B* **267**, 2267-2272. (doi:10.1098/rspb.2000.1278).

105. Slater GJ. 2015 Iterative adaptive radiations of fossil canids show no evidence for diversity-dependent trait evolution. *Proc. Natl. Acad. Sci. U. S. A.* **112**, 4897-4902. (doi:10.1073/pnas.1403666111).

106. Paradis E. 2006 *Analysis of Phylogenetics and Evolution with R*. New York, Springer; 211 p.

107. Harmon LJ, Weir J, Brock C, Glor RE, Challenger W. 2008 GEIGER: investigating evolutionary radiations. *Bioinformatics* **24**, 129-131. (doi:doi:10.1093/bioinformatics/btm538).

108. Hurvich CM, Simonoff JS, Tsai CL. 1998 Smoothing parameter selection in nonparametric regression using an improved Akaike Information Criterion. *J. R. Stat. Soc. B* **60**, 271–293.
